# Return of a giant: DNA from archival museum samples helps to identify a unique cutthroat trout lineage formerly thought to be extinct

**DOI:** 10.1098/rsos.171253

**Published:** 2017-11-15

**Authors:** Mary M. Peacock, Evon R. Hekkala, Veronica S. Kirchoff, Lisa G. Heki

**Affiliations:** 1 Department of Biology, University of Nevada, Reno, NV 89557, USA; 2 Ecology, Evolution, and Conservation Biology Interdisciplinary Program, University of Nevada, Reno, NV 89557, USA; 3 Department of Biological Sciences, Fordham University, New York, NY 10458, USA; 4 United States Department of Agriculture, Agricultural Research Service, Reno, NV 89512, USA; 5 United States Fish and Wildlife Service, Lahontan National Fish Hatchery Complex, 1340 Financial Blvd, Suite 234, Reno, NV 89502, USA

**Keywords:** Bayesian clustering analysis, Lahontan cutthroat trout, *Oncorhynchus clarkii henshawi*, microsatellites, museum specimens, archival DNA

## Abstract

Currently one small, native population of the culturally and ecologically important Lahontan cutthroat trout (*Oncorhynchus clarkii henshawi*, LCT, Federally listed) remains in the Truckee River watershed of northwestern Nevada and northeastern California. The majority of populations in this watershed were extirpated in the 1940s due to invasive species, overharvest, anthropogenic water consumption and changing precipitation regimes. In 1977, a population of cutthroat trout discovered in the Pilot Peak Mountains in the Bonneville basin of Utah, was putatively identified as the extirpated LCT lacustrine lineage native to Pyramid Lake in the Truckee River basin based on morphological and meristic characters. Our phylogenetic and Bayesian genotype clustering analyses of museum specimens collected from the large lakes (1872–1913) and contemporary samples collected from populations throughout the extant range provide evidence in support of a genetically distinct Truckee River basin origin for this population. Analysis of museum samples alone identified three distinct genotype clusters and historical connectivity among water bodies within the Truckee River basin. Baseline data from museum collections indicate that the extant Pilot Peak strain represents a remnant of the extirpated lacustrine lineage. Given the limitations on high-quality data when working with a sparse number of preserved museum samples, we acknowledge that, in the end, this may be a more complicated story. However, the paucity of remnant populations in the Truckee River watershed, in combination with data on the distribution of morphological, meristic and genetic data for Lahontan cutthroat trout, suggests that recovery strategies, particularly in the large lacustrine habitats should consider this lineage as an important part of the genetic legacy of this species.

## 1. Introduction

**Figure 1.**
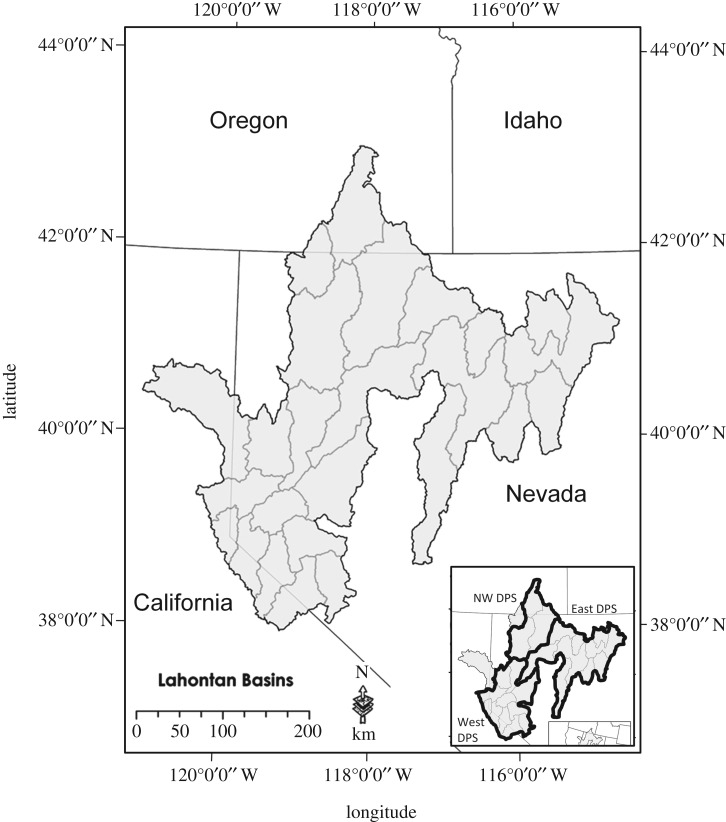
The Lahontan hydrographic basin with all major watersheds outlined and the three distinct population segments indicated in bold in the inset.

The integrity of freshwater ecosystems is threatened by invasive species, human water consumption and changing precipitation regimes. Freshwater fishes, particularly those from arid regions, are among the most imperiled taxa worldwide. In the western United States, cold water salmonids are especially vulnerable and many have been extirpated from substantial portions of their historical range [1–3]. The inland cutthroat trout subspecies (*Oncorhynchus clarkii* spp.) are cold water fishes that were heavily impacted by European expansion into western North America, with many now listed as threatened or endangered under the United States Endangered Species Act (ESA). These fishes were once found throughout the intermontane western USA in multiple-order stream and lake habitats. Habitat losses due to the introduction of non-native trout, water diversions, water pollution and riparian habitat degradation have largely isolated cutthroat trout populations into small headwater reaches or led to local extirpations outright [4–12]. Both the demographic and environmentally stochastic processes which are characteristic of small populations increasingly threaten these unique lineages [9]. Here we report on genetic results that support the rediscovery of a lacustrine form, long thought to be extinct, of Lahontan cutthroat trout (*O. clarkii henshawi*, LCT), an ESA-listed (USFWS Federal Register vol. 40, p. 29 864) native to the Lahontan hydrographic basin of northern Nevada, northeastern California and southeastern Oregon, in waters outside the native range (figure 1).

### 1.1. Lahontan cutthroat trout background

**Figure 2.**
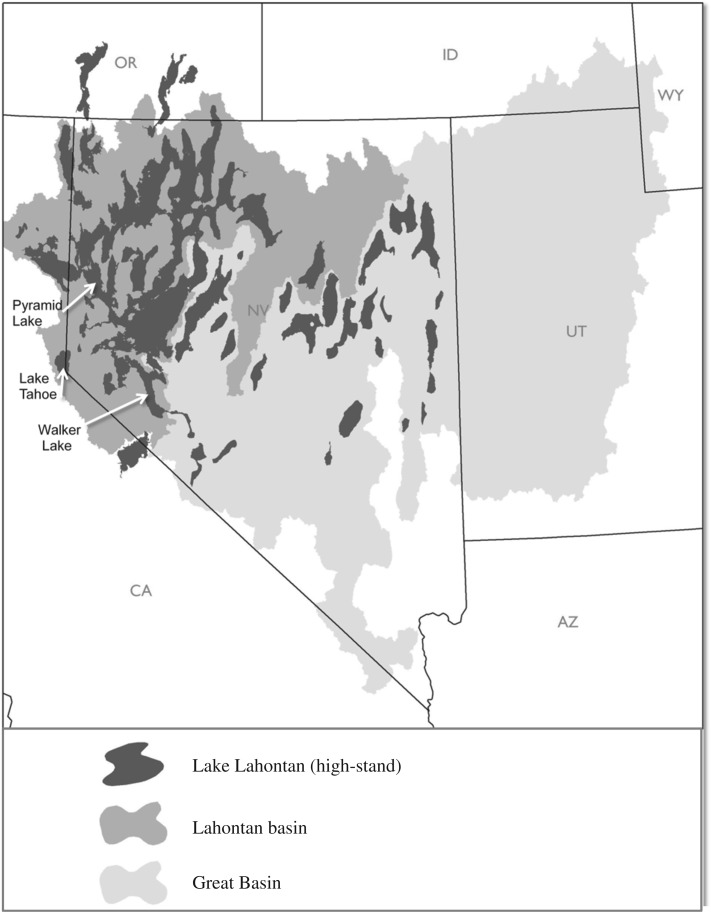
The high stand of pluvial Lake Lahontan that occurred approximately 650 kyr BP is indicated by black shading. Endorheic Pyramid and Walker Lakes, remnants of the pluvial lake are labelled along with oligotrophic Lake Tahoe (Figure by Matt Mayfield, Trout Unlimited).

**Figure 3.**
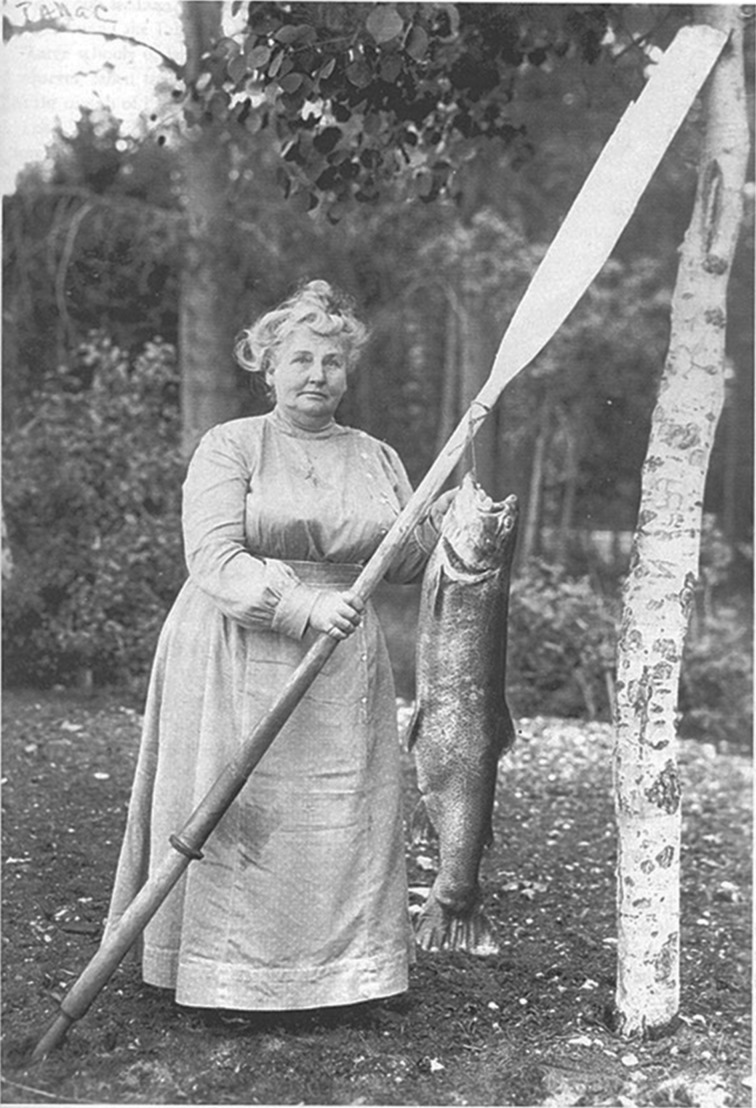
Late nineteenth century photo of Lahontan cutthroat trout caught in Lake Tahoe. Photo courtesy of Jim Bell and Velma Comstock Eden collection.

The Lahontan cutthroat trout evolved in pluvial Lake Lahontan, a large endorheic lake which reached its high stand 650 kyr BP during the Pleistocene, and covered much of northwestern Nevada (figure 2; [13]). The lake ebbed and flowed over time, desiccating to near present-day levels about 8000 years ago [14]. Contemporary Pyramid and Walker lakes are remnants of the ancient pluvial lake and retained their Lahontan cutthroat trout fisheries until human-mediated extirpations occurred in the mid-twentieth century (figure 2). The Lahontan cutthroat trout native to Lake Tahoe, Pyramid and Walker lakes had high growth rates and were considered the largest inland trout in North America prior to extirpation and provided a primary resource to Native American communities in the region (figure 3; [15,16]).

Three evolutionary significant units (ESUs) or distinct population segments (DPSs) have been identified for Lahontan cutthroat trout based on morphological, meristic, genetic and ecological data: (1) western Lahontan basin—Truckee, Carson and Walker rivers watersheds, which contain the only large lacustrine habitats; (2) eastern Lahontan basin—Humboldt and Reese rivers watersheds; and (3) northwestern Lahontan basin—Quinn River, Coyote Lake and Summit Lake basins (figure 1 inset; [17]). The ESU concept [18–20], which was envisaged as a way to delineate evolutionary lineages within species in order to optimize conservation strategies, has been widely employed to distinguish genetic and ecologically distinct groups of populations for many salmonid species including inland trout species[21–25].

**Figure 4.**
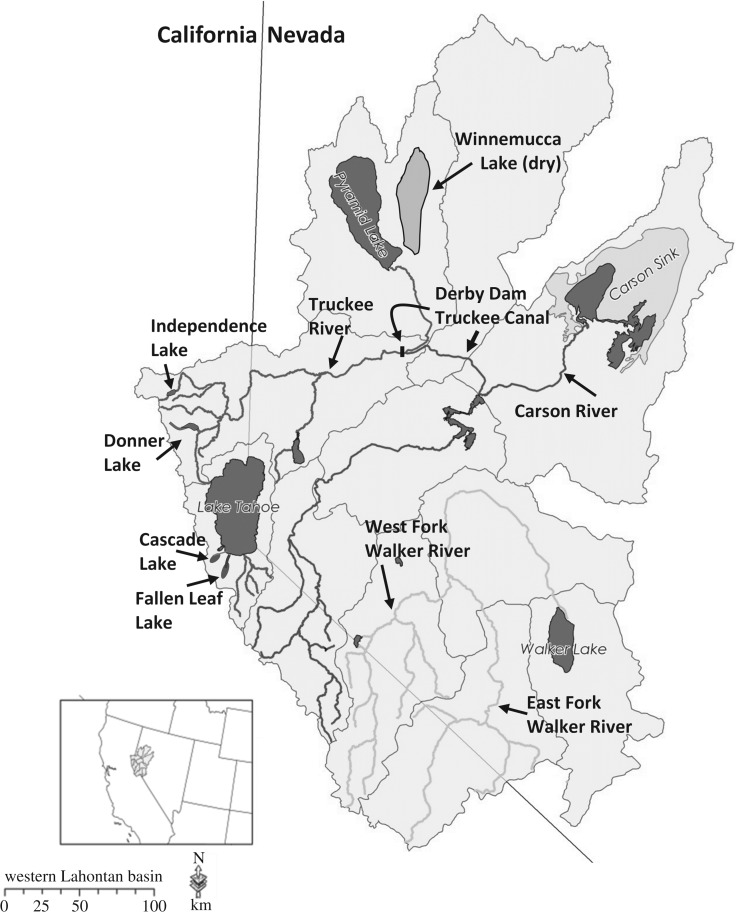
The western Lahontan basin ESU comprised the Truckee, Carson and Walker river watersheds with the main stem rivers and all lacustrine habitats indicated. Derby Dam constructed in 1904 is a water diversion dam on the lower Truckee River, which isolates fish in the lower watershed. Winnemucca Lake is now a dry lake bed due to water diversions from the Truckee River into the Truckee Canal at Derby Dam and subsequent flow reductions in the lower river in the early part of the twentieth century.

Lahontan cutthroat trout have been lost from more than 90% of their historical habitat and, as with the other cutthroat trout subspecies, are largely found in small, isolated headwater reaches in ≤10 km of habitat [4,17]. The western Lahontan basin has sustained the greatest losses with more than 99% of populations lost from historical habitat, with the lacustrine form native to these waters having been largely extirpated (figure 4). Lahontan cutthroat trout were once found throughout the Truckee River basin where most of the large lacustrine habitat is found (i.e. Lake Tahoe, Pyramid and Winnemucca lakes and four smaller lakes in the upper watershed: Cascade, Donner, Fallen Leaf and Independence lakes). The Truckee River, which flows 195 kilometres connects oligotrophic Lake Tahoe to endorheic Pyramid Lake (figure 4). Today the Truckee River watershed has one native extant population, a small, naturally reproducing lacustrine population (Independence Lake) in the upper watershed. A steep decline in Pyramid Lake elevation ensued after 1910 following the construction of Derby Dam and the Truckee Canal in the lower river that diverted Truckee River water to the Newlands Irrigation Project in the Carson River basin (see figure 4), preventing access to the fluvial spawning habitat and resulting in the complete dry down of adjoining shallow Winnemucca Lake and complete loss of the fishery by the 1940s. Lake Tahoe also lost its population in the mid-twentieth century primarily due to the introduction of non-native salmonids, overharvest and loss of spawning habitat.

Prior to the extirpation of the Lahontan cutthroat trout from Lake Tahoe, the Truckee River and Pyramid Lake, cutthroat trout from this watershed were used to augment existing and to create de novo populations throughout the Lahontan basin [26]. Records on specific locations and success of these transplants were, however, not generally well kept (Nevada Division of Wildlife records, NDOW), but genetic data indicate that these transplants were largely unsuccessful [27–30]. During this time period, cutthroat trout were stocked into the fishless Morrison Creek drainage in the Pilot Peak Mountains in the Bonneville hydrographic basin of Utah on the border with Nevada, where they have persisted to this day. Three phenotypic characters consistent with a lacustrine life history putatively supported a western Lahontan basin origin for these cutthroat trout: (i) uniform distribution of moderately large, roundish spots over the sides of the body and ventral surface, (ii) the large number of gill rakers (21–28; modal and mean values typically 23–25 versus 17–21 in other subspecies), and (iii) the large number of pyloric caeca (typically 45–80 versus 25–50 in other subspecies) [26]. The greater number of gill rakers in lacustrine fish facilitates plankton feeding in lake habitats and increased number of pyloric caeca tends to be associated with piscivory among salmonines [20]. Based on these characteristics, Hickman and Behnke [26] suggested that these trout could be the original Pyramid Lake strain and trout from Morrison Creek were subsequently translocated into neighbouring Bettridge Creek in order to expand the population. Trout from Morrison Creek were brought into the United States Fish and Wildlife Service (USFWS) Lahontan National Fish Hatchery in 1995 to be used for recovery activities specifically in lacustrine habitat in the Truckee River basin. Based upon the morphological and meristic data for the Pilot Peak strain, which support a lacustrine life history, the USFWS and the Pyramid Lake Paiute tribe began transplanting the Pilot Peak strain into Pyramid Lake, historical lake habitat found on tribal lands in 2006 (USFWS, Pyramid Lake Paiute tribe personal communication).

After the extirpation of the original fishery, a hatchery-maintained fishery of mixed-stock origin was established (the contemporary Pyramid Lake strain), which included trout from Independence Lake, Summit Lake and the Carson River [31]. However, the mixed-stock contemporary strain never achieved the historical large sizes in the Pyramid Lake environment, whereas the Pilot Peak strain is now achieving very large sizes in Pyramid Lake (≥11 kg; https://www.fws.gov/lahontannfhc/), reminiscent of the historical population, which produced the largest cutthroat trout on the globe. The Pilot Peak cutthroat trout are also piscivorous, which has been observed in the both the hatchery and in Pyramid Lake (personal communication, Tim Loux, USFWS Lahontan National Fish Hatchery Complex).

In addition to the morphological data, evidence from earlier genetic studies [29] and some very recent work (post our analyses) based on RADseq SNP data [32] have verified that the Pilot Peak cutthroat trout are Lahontan cutthroat trout. Genetic determination of the origin of the Pilot Peak trout using known Truckee River museum specimens, however, was not undertaken until this study.

### 1.2. Study objectives

We chose microsatellite markers for this current work, as both mtDNA sequence and RFLP data from earlier analyses [27,29] were not variable enough to resolve within ESU/DPS relationships and so were unable to answer the questions we were asking, especially relationships at the multiple spatial scales we were examining. Here, we compare genetic data generated for six polymorphic nuclear microsatellite markers developed from contemporary Lahontan cutthroat trout [33], for museum specimens of Lahontan cutthroat trout collected between 1872 and 1913 from Lake Tahoe, the Truckee River and Pyramid Lake (table 1) with fish collected from Morrison and Bettridge creeks and Lahontan cutthroat trout populations sampled from the majority of currently occupied drainages.

**Table 1.** Lahontan cutthroat trout samples collected from Lake Tahoe, California and the Truckee River and Pyramid Lake, Nevada in 1872–1913. Museum, sampling location, year of collection and collector are included for each sample. Curators at each museum provided us with muscle and/or skin tissue from the individual cutthroat trout listed here. See figure 1 for location information.

museum collection	sampling location	year	collector
*California Academy of Sciences* (*N = 8*)			
CAS-SU 13298	Truckee River—near Derby	1911	J.O. Snyder
CAS-SU 13299	Truckee River—near Derby	1911	J.O. Snyder
CAS-SU 13300	Truckee River—near Derby	1911	J.O. Snyder
CAS-SU 13301	Truckee River—near Derby	1911	J.O. Snyder
CAS-SU 13302	Pyramid Lake, the Willows	1911	J.O. Snyder
CAS-SU 13303	Truckee River—near Derby	1911	J.O. Snyder
CAS-SU 13304	Lake Tahoe	1911–1913	J.O. Snyder
CAS-SU 13305	Lake Tahoe	1911–1913	J.O. Snyder
*Smithsonian Institution* (*N = 53*)			
USNM 015496	Lake Tahoe—location unknown	1872	L. Stone
USNM 017085	Lake Tahoe—location unknown	1876	H.W. Henshaw
USNM 017086	Lake Tahoe—location unknown	1876	H.W. Henshaw
USNM 021083	Lake Tahoe—location unknown	1876	H.W. Henshaw
USNM 023467	Lake Tahoe—location unknown	1876	H.W. Henshaw
USNM 075165	Lake Tahoe—near Tallac	1911	J.O. Snyder
USNM 075166	Lake Tahoe—near Tallac	1911	J.O. Snyder
USNM 075167	Lake Tahoe—near Tallac	1911	J.O. Snyder
USNM 075168	Lake Tahoe—near Tallac	1911	J.O. Snyder
USNM 075169	Lake Tahoe—near Tallac	1911	J.O. Snyder
USNM 075170	Lake Tahoe—near Tallac	1911	J.O. Snyder
USNM 075171	Lake Tahoe—near Tallac	1911	J.O. Snyder
USNM 075172	Lake Tahoe—near Tallac	1911	J.O. Snyder
USNM 075173	Lake Tahoe—near Tallac	1911	J.O. Snyder
USNM 075174	Lake Tahoe—near Tallac	1911	J.O. Snyder
USNM 075175	Lake Tahoe—near Tallac	1911	J.O. Snyder
USNM 075187	Lake Tahoe—location unknown	1876	H.W. Henshaw
USNM 075188	Lake Tahoe—location unknown	1876	H.W. Henshaw
USNM 075189	Lake Tahoe—location unknown	1911	J.O. Snyder
USNM 075190	Lake Tahoe—location unknown	1911	J.O. Snyder
USNM 075191	Lake Tahoe—location unknown	1911	J.O. Snyder
USNM 075192	Lake Tahoe—location unknown	1911	J.O. Snyder
USNM 075193	Lake Tahoe—location unknown	1911	J.O. Snyder
USNM 075194	Lake Tahoe—location unknown	1911	J.O. Snyder
USNM 075195	Lake Tahoe—location unknown	1911	J.O. Snyder
USNM 075196	Lake Tahoe—location unknown	1911	J.O. Snyder
USNM 075197	Lake Tahoe—location unknown	1876	H.W. Henshaw
USNM 107328	Lake Tahoe—location unknown	1876	H.W. Henshaw
USNM 075176	Truckee River—near Derby	1911	J.O Snyder
USNM 075178	Truckee River—near Derby	1911	J.O. Snyder
USNM 075179	Truckee River—near Derby	1911	J.O. Snyder
USNM 075180	Truckee River—near Derby	1911	J.O. Snyder
USNM 075181	Truckee River—near Derby	1911	J.O. Snyder
USNM 075206	Truckee River—near Derby	1911	J.O. Snyder
USNM 075208	Truckee River—near Derby	1911	J.O. Snyder
USNM 075209	Truckee River—near Derby	1911	J.O. Snyder
USNM 075210	Truckee River—near Derby	1911	J.O. Snyder
USNM 075211	Truckee River—near Derby	1911	J.O. Snyder
USNM 075212	Truckee River—near Derby	1911	J.O. Snyder
USNM 075213	Truckee River—near Derby	1911	J.O. Snyder
USNM 075214	Truckee River—near Derby	1911	J.O. Snyder
USNM 075182	Pyramid Lake—location unknown	1911	J.O. Snyder
USNM 075183	Pyramid Lake—location unknown	1911	J.O. Snyder
USNM 075184	Pyramid Lake—location unknown	1911	J.O. Snyder
USNM 075185	Pyramid Lake—location unknown	1911	J.O. Snyder
USNM 075198	Pyramid Lake—location unknown	1911	J.O. Snyder
USNM 075199	Pyramid Lake—location unknown	1911	J.O. Snyder
USNM 075200	Pyramid Lake—location unknown	1911	J.O. Snyder
USNM 075201	Pyramid Lake—location unknown	1911	J.O. Snyder
USNM 075202	Pyramid Lake—location unknown	1911	J.O. Snyder
USNM 075203	Pyramid Lake—location unknown	1911	J.O. Snyder
USNM 075186	Winnemucca Lake	1911	J.O. Snyder
USNM 075705	Winnemucca Lake	1911	J.O. Snyder
*University of Michigan (N = 2)*			
UM 176347	Cascade Creek	1913	J.O. Snyder
UM 176347	Cascade Creek	1913	J.O. Snyder

We asked a number of questions on these temporally and spatially stratified genetic data including: (i) do the data support a Truckee River basin origin for Morrison Creek (Pilot Peak strain) Lahontan cutthroat trout; and if so (ii) have the Morrison Creek trout retained levels of genetic variation that are comparable to the historic Truckee River basin samples; (iii) how similar genetically are contemporary Independence Lake Lahontan cutthroat trout to both the Morrison Creek and Truckee River basin museum samples; and finally (iv) how can these data inform recovery of Lahontan cutthroat trout in the western basin ESU?

## 2. Material and methods

### 2.1. Samples

#### 2.1.1. Museum-preserved specimens

Tissue samples were obtained from 62 museum specimens from vertebrate collections at the California Academy of Sciences (CAS), the Smithsonian Institution (USNM) and University of Michigan (UMMZ). These collections included Lahontan cutthroat trout collected from Lake Tahoe (*N* = 31), the lower Truckee River at Derby Dam (*N* = 18) and Pyramid and Winnemucca lakes (*N* = 13) (1872–1913) (table 1). All tissue samples were originally preserved in formalin but were transferred to ethanol by museum personnel at some point after the original collection. We received all samples stored in 70% ethanol. In our experience, successful DNA extraction for formalin-fixed samples varies greatly depending on original collector, duration of fixation and size of specimen. Tissues from larger specimens tend to be less perfused. Samples were kept separate from areas in which prior work on salmonids had occurred, and all protocols for extraction and amplification followed clean-room techniques as in Hekkala *et al*. [34,35].

#### 2.1.2. Extant populations

Fin clips were collected from adult Lahontan cutthroat trout in Morrison and Bettridge creeks and the Pilot Peak hatchery broodstock by USFWS fisheries biologists. Samples from extant, native Lahontan cutthroat trout populations from the three designated ESUs were provided by the Peacock laboratory at the University of Nevada (UNR), Trout Unlimited, California, Nevada and Oregon state game and fish agencies as well as United States federal agencies (USFWS, US Forest Service and Bureau of Land Management) (table 2). The Pyramid Lake Paiute tribe supplied fin clip samples from their hatchery-maintained contemporary mixed-stock strain.

**Table 2.** Extant Lahontan cutthroat trout populations included in this study listed by ESU and river basin. *N* is the number of individuals sampled per population for this study. Abbreviations for each population that are used elsewhere in the document are listed.

ESU/DPS	population	abbreviation	*N*	year sampled
Western basin				
Truckee River basin				
	Independence Lake	INL	20	2002
	Heenan Lake (INL origin)	HEL	47	2002
	contemporary Pyramid Lake	CPYR	34	2002
	Truckee River historical samples	TRM	62	
	Lake Tahoe		32	1872–1913
	Cascade	—	8	1911–1913
	Tahoe	—	8	1872–1876
	Tahoe	—	4	1911–1913
	Tallac	—	11	1913
	Lower Truckee River (Derby Dam)		18	1911
	Pyramid Lake	PYL	11	1911
	Winnemucca Lake	WIN	2	1911
Carson River basin				
	East Carson River	CAR	42	2001
	Murray Creek	MUC	20	2001
	Poison Flat Creek	POC	38	2001
	Pacific Valley River	PAC	30	2001
	Milk Ranch Creek	MIC	35	2001
	Marshall Canyon Creek	MAC	42	2001
Walker River basin				
	By-Day Creek	BDC	29	2001
	Slinkard Creek	SLC	38	2001
	Wolf Creek	WOC	30	2001
	Mill Creek	MILL	30	2001
	Silver Creek	SILV	6	2001
Northeastern basin				
	Coyote Lake sub-basin			
	Little Whitehorse Creek	LW	34	1996
	Whitehorse Creek	WH	15	1996
	Willow Creek	WC	37	1996
	Cottonwood Creek	CW	9	1996
	Quinn River basin			
	Washburn Creek	WAC	24	1997
	Crowley Creek	CRC	36	1997, 2002
	Line Canyon Creek	LIC	28	2000
Eastern basin				
Humboldt River basin				
	Little Humboldt River sub-basin			
	Abel Creek	ABC	36	2000
	Indian Creek	INC	34	
	Rock Creek sub-basin			
	Frazer Creek	FRC	55	1996, 1997, 2000
	Maggie Creek sub-basin			
	Beaver Creek	BVC		
	Coyote Creek	COY	55	2002
	Little Jack Creek	L J	53	2001
	North Fork Humboldt River sub-basin		38	2001
	Foreman Creek	FORE	24	2000
	Gance Creek	GAC	26	2000
	North Fork Humboldt River	NFH	48	1996, 2000
	Marys River sub-basin			
	East Marys River	EMR	36	2000
	West Marys River	WMR	48	1998, 2000
	Reese River sub-basin			
	Mohawk Creek	MHK	33	1996–2000
	Tierney Creek	TIC	30	2000
Out-of-basin	*putative Truckee River basin*			
Pilot Peak Mtns Utah	Morrison Creek	MOC	31	2001
	Bettridge Creek	BEC	29	2001
	Pilot Peak LNFH broodstock	Pilot	22	2014

### 2.2. Extraction of DNA

#### 2.2.1. Museum-preserved samples

Approximately 25 mg of tissue was removed from each sample and placed in 1.0 ml of phosphate-buffered saline (PBS) solution and incubated at room temperature for one hour. Samples were inverted frequently to wash the preservatives off the sample. The samples were washed twice, and the tissues were then transferred to 360 µl of ATL, a tissue lysis buffer provided in the Qiagen DNA extraction kit, and 40 µl of proteinase K incubated minimally overnight at 55°C to ensure complete digestion of the tissue. This process was repeated twice. After digestion 400 µl of the AL buffer provided in Qiagen kits was added and the samples inverted rapidly for 15 s to yield a homogeneous solution. DNA precipitation was carried out by adding an equal volume of cold 100% ethanol to the lysate. Samples were mixed thoroughly and incubated at 4°C for at least one hour. The ethanol/lysate mix was transferred to Qiagen DNeasy columns and centrifuged at 6000*g* for one minute to bind the DNA to the filter in the column. Columns were washed twice with Qiagen AW1 and AW2 buffers and allowed to dry. A 50 μl aliquot of elution buffer heated to 70°C was applied to the column and incubated at room temperature for one hour. A second elution step was carried out using the same protocol. This process yielded 100 μl of genomic DNA.

#### 2.2.2. Contemporary samples

DNA was extracted from fin clips collected during field surveys from extant populations as in Peacock *et al*. [25]. Genomic DNA was extracted using Qiagen DNeasy tissue kits as per the manufacturer's guidelines and then quantified per individual using a Labsystems Fluoroskan Ascent fluorometer.

### 2.3. Genetic markers

We tested 18 microsatellite loci developed for Lahontan cutthroat trout for use with historical museum-preserved specimens and used six of these loci (OCH5, OCH6, OCH9, OCH15, OCH16 and OCH17; [33]) that consistently amplified DNA isolated from the preserved samples and have been used to characterize genetic variation in over 50 Lahontan cutthroat trout populations [25,30,36–38].

### 2.4. Polymerase chain reactions

All polymerase chain reactions (PCRs) were carried out on an MBS Satellite 0.2G thermal cycler in 16 µl volumes. We used multiplex (3–4 microsatellite primer pairs) PCRs per individual for contemporary samples. For museum samples we used both individual locus and two loci PCRs. The PCR conditions for all loci OCH5–OCH17 can be found in Peacock *et al*. [33]. PCR product was diluted in deionized water to an appropriate intensity determined by dilution tests and 1 µl of the product was added to 19 µl of GeneScan 500 LIZ size standard with Hi-Dye formamide (ABI, Perkin-Elmer Corporation). Fragment analysis was carried out on an ABI Prism 3730 DNA analyser. Alleles were scored using the ABI Prism GeneScan (v. 3.5.1) and GeneMapper (v. 3.0) software (ABI).

### 2.5. Museum samples and genotyping errors

Although no systematic patterns of null alleles or allelic dropout have been documented for these loci in extant populations, we used Micro-checker (v. 2.2.3, [39]) to test for null alleles and allelic dropout per locus for the Truckee River museum samples, and Morrison, Bettridge, Independence and Heenan lake (derived from Independence Lake) populations.

To minimize genotyping error rate for the museum-preserved samples, we used the multiple tubes method of Taberlet *et al*. [40]. Initially, four independent PCR reactions were carried out per individual museum sample for all loci. The resulting traces were compared and a consensus genotype was called per locus per individual if three of four reactions yielded consistent results. An additional four PCRs were run for those individuals for which we could not call a consensus genotype from the first four PCRs. If the results from the second four independent PCR reactions were still inconsistent or the PCR failed after eight replicates, we did not generate a genotype at that locus for those individuals. We included negative controls through the extraction and PCR protocols and confirmed at each step that no contamination from modern samples was present.

We calculated an allelic dropout rate using the equation ADO_μ_ = *D_j_*/*A*_hetj_, where *D*_j_ is the number of amplifications where a dropout event was observed and *A*_hetj _ is the number of amplifications of heterozygotes. The number of false alleles (FA_μ_) was calculated as FA_μ_ = *F*_j_/*A*_j_, where *F*_j_  is the number of amplifications at locus j where a false allele is observed, and *A*_j_  is the total number of amplifications of both heterozygotes and homozygotes (notation after Lambert *et al*., [41]).

### 2.6. Statistical analysis

#### 2.6.1. Genetic variation

We used FSTAT (v. 2.9.3.2; [42]) to quantify the number of alleles (*N*_A_) and the allelic richness (*R*_S_) per locus, estimate average levels of observed and expected heterozygosity (Ho, He), and test for deviations from the Hardy–Weinberg equilibrium (HWE) over all loci and populations. We tested for genetic bottlenecks in the Morrison, Bettridge, Independence and Heenan lake populations as well as the Truckee River basin museum samples using the heterozygous excess method, the two-phase (TPM) and single-step (SMM) mutation models, and the Wilcoxon-signed rank test in the program BOTTLENECK (v. 1.2.0; [43]).

#### 2.6.2. Analysis of molecular variance and principal coordinates analysis

Analysis of molecular variance (AMOVA) and principal coordinates analysis (PCoA) were conducted in GenAlEx 6.5 [44,45]. We conducted AMOVA in order to characterize how genetic variation was partitioned within and among populations. PCoA was used to examine how genetic variation within the museum samples was distributed within the Truckee River watershed and to further examine the relationship between the Pilot Peak strain and the museum samples.

#### 2.6.3. Population genetic structure

We calculated pairwise F_ST_ using GenAlEx 6.5: (i) among river basins, where all populations were grouped by river of origin, and (ii) among sites within the Truckee River basin (museum sampling sites, contemporary Pilot Peak strain and Independence Lake). We also used a Bayesian genotype clustering approach to assess genetic structure among populations within and among watersheds in a range-wide analysis including the Truckee River basin museum samples (STRUCTURE 2.3.4; [46]). In a separate Bayesian analysis of the Truckee River watershed, we compared the Pilot Peak strain and Independence Lake to the museum samples. We also conducted a Bayesian analysis of the museum samples only. The grouping criteria in STRUCTURE include HWE and gametic phase equilibrium between loci within groups. We used an admixture model and specified a range of 1–15 potential genotype clusters (*k*) for the range-wide analysis, 1–8 for the Truckee River contemporary and museum samples, and 1–6 for the museum samples only analysis. We specified a 1 000 000 iteration burn-in period followed by ten 1 000 000 Markov chain Monte Carlo replicates per *k* to approximate posterior allelic distributions against which individual genotypes were compared and assigned to a cluster for both range-wide and Truckee River basin analyses [46]. We used the Δ*k* method of Evanno *et al*. [47] to determine the optimal *k*.

#### 2.6.4. Phylogenetic analyses

We constructed a number of phylogenetic trees using a Cavalli-Sforza distance metric and neighbour-joining tree building algorithm in the Populations 1.2.26 (1000 replications; [48]) and visualized in TreeView [49]. Populations were grouped by river for a range-wide analysis, by contemporary and museum samples within the Truckee River analysis, and by museum sampling locations within the Truckee River watershed.

## 3. Results

### 3.1. Museum samples genotyping

We were able to reliably genotype 24 of the museum samples at 6 of the 6 loci, 15 at 5 loci, 18 at 4 loci and 5 samples at 3 loci. The allelic dropout rate (ADO_μ_) was greatest for the OCH5 locus (0.31; table 3). The percentage of false alleles (FA_μ_) was highest for OCH17 (0.22).

**Table 3.** Allele size range (bp), global observed (Ho) and expected (He) heterozygosities, and genotyping errors at six microsatellite loci amplified from DNA isolated from museum-preserved samples of Lahontan cutthroat trout. ADOμ (total allelic dropout rate), no. of times the allele was missing/number of amplifications that were determined to be heterozygous. FAμ (false allele rate), no. of times an extra or different allele was found/ total scorable PCRs.

locus	OCH5	OCH6	OCH9	OCH15	OCH16	OCH17
allele size range (bp)	178–269	161–230	162–229	302–380	188–228	163–307
Ho	0.792	0.544	0.443	0.889	0.827	0.600
He	0.848	0.76	0.613	0.855	0.815	0.921
allelic dropout longer allele missing^a^	6	9	5	5	4	2
allelic dropout shorter allele missing^b^	13	3	4	0	0	0
ADO_μ_	0.31	0.24	0.29	0.06	0.06	0.10
FA_μ_	0.04	0.01	0.14	0.09	0.15	0.22

^a^Allelic dropout longer allele missing = no. of times a longer allele was missing/number of amplifications that were determined to be heterozygous.

^b^Allelic dropout shorter allele missing =no. of times a shorter allele was missing/number of amplifications that were determined to be heterozygous.

### 3.2. Genetic variation

The average observed heterozygosity (Ho) over all loci ranged from 0.171–0.833 among the extant populations. Ho of the extant western Lahontan basin populations in the Truckee, Carson and Walker rivers and the Pilot Peak strain were 0.683, 0.411, 0.267 and 0.488, respectively. The Truckee River basin museum samples had relatively high Ho (0.720–0.833). Bettridge and Morrison (Pilot) creek populations had fewer alleles per locus compared to the Truckee River basin museum samples and Independence Lake (TRM, *N*_A_* *= 9–16; INL = 2–13; MOC&BEC, *N*_A_* *= 2–6) and lower allelic richness (TRM, *R*_S_ = 7.62–10.6; MOC&BEC, *R*_S_ = 2–5.66), but similar *N*_A_ and *R*_S_ compared to other extant LCT populations in the Humboldt (e.g. NFH, *N*_A_ = 5–7, *R*_S_ = 3.4–4.9; LJC, *N*_A_ = 3, *R*_S_ = 2.3– 2.9), Quinn (WAC *N*_A_ = 2–4, *R*_S_ = 2–3.25) and Willow-Whitehorse rivers (WWH-CW *N*_A_ = 2–6, *R*_S_ = 2–6).

Significant positive *F*_IS_ values were found at single loci in multiple populations in the Humboldt and Quinn rivers, but no locus was out of HWE in every population and no population had significant *F*_IS_ values for all loci [*p* = 0.00042, adjusted *p* value based on 2400 randomizations; OCH5, COY (Maggie Creek sub-basin), *F*_IS_ = 0.384; OCH6, FRC (Rock Creek sub-basin), *F*_IS_ = 0.352; and OCH15, CRC (Quinn River basin), *F*_IS_ = 0.513].

There was no evidence of null alleles in the Bettridge, Morrison, Independence and Heenan lake populations or in the museum samples. The Morrison Creek population was the only population of those tested (Morrison, Bettridge, Independence, Heenan and the museum samples) that showed evidence of a bottleneck under both the TPM and SMM mutation models (*p* < 0.04). In a previous analysis [30], bottlenecks were observed for the Carson and Walker river populations (*p* ≤ 0.005) and multiple populations within the Humboldt (i.e. Mohawk and Tierney, *p* ≤ 0.005) and Quinn rivers (Crowley, Line Canyon and Washburn, 0.005 < *p* < 0.05).

### 3.3. Population genetic structure

#### 3.3.1. Range-wide analysis: Pilot Peak LCT and river basin of origin

**Figure 5.**
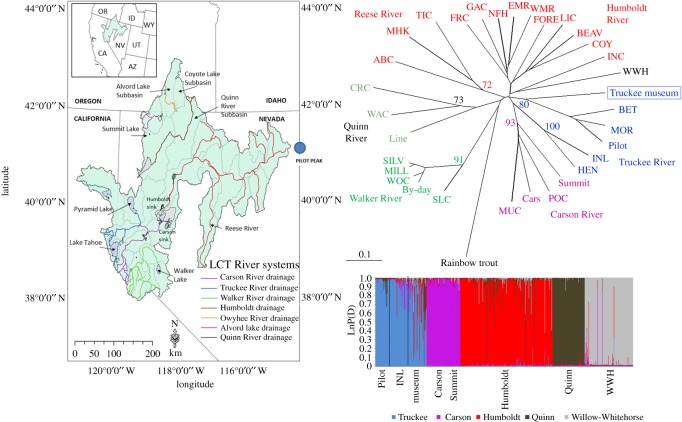
Lahontan hydrographic basin (shaded) with rivers colour coded, and Pilot Peak location on the border with Utah indicated with a blue circle. Bayesian genotype clustering analysis (LnP(D) = −1157.78, *k* = 5, Δ*k* = 3.95) and Cavalli-Sforza genetic distance and neighbour-joining phylogenetic tree (bootstrap values indicated at nodes) for all contemporary populations sampled and the Truckee River basin historical museum samples. Watersheds are colour coded as follows: Truckee River basin including museum samples, blue; Carson River and Summit Lake, purple; Humboldt, red; Quinn River, brown; Willow-Whitehorse (WWH), grey.

Genetic variation was partitioned primarily within individuals across the range of Lahontan cutthroat trout (both contemporary and museum samples) with 61% within individuals, 20% among individuals, 17% among populations and only 2% among regions (the designated ESUs). There was also substantial genetic structure range wide as all pairwise *F*_ST_ estimates among river basins were significant (*p* = 0.01, 99 permutations; table 4*a*). In the river-based Bayesian analysis, *k* = 5 had the highest statistical support (LnP(D) = −1157.78, Δ*k* = 3.95) and individuals were assigned primarily to single genotype clusters that overlapped with major river drainage of origin (figure 5). The Pilot Peak strain (BET, MOR and Pilot hatchery), Independence Lake and the Truckee River basin museum samples were assigned to the same genotype cluster (blue). Several individuals from the museum samples had high proportional membership in the ‘red’ Humboldt River genotype cluster, which probably reflects the fluctuating history of the pluvial lake and periodic inundation of some of the Humboldt River watersheds [13]. Individuals in Summit Lake, while physically in the northwestern Lahontan basin ESU, were assigned to the same genotype cluster as the Carson River populations, which also reflects the pluvial lake connections during the Pleistocene [13]. The topology of the Cavalli-Sforza river-based neighbour-joining tree largely mirrored the Bayesian genotype cluster results (figure 5); however, here the Truckee River museum samples formed a clade with the Pilot Peak strain (BET, MOR, Pilot hatchery) with relatively high bootstrap support (80%), while Independence Lake (INL and HEN) formed a clade with the Carson River populations, but with low bootstrap support (42%; figure 5).

**Table 4.** Pairwise *F*_ST_ estimates among all major watersheds (*a*) and among Truckee River basin historical museum sampling locations (*b*). All pairwise *F*_ST_ estimates are significant (*p* = 0.01, 99 permutations).

(*a*)	Truckee (Pilot&BET,MOR)	museum	Carson	Humboldt	Quinn
Truckee					
museum	0.110				
Carson	0.200	0.205			
Humboldt	0.183	0.185	0.297		
Quinn	0.331	0.307	0.446	0.426	
Willow-Whitehorse	0.247	0.198	0.335	0.294	0.444

(*b*)	Pilot (BET, MOR)	Independence	Tahoe museum	Truckee museum	Pyramid Lake museum
Pilot					
Independence	0.240				
Tahoe museum	0.167	0.122			
Truckee museum	0.260	0.194	0.058		
Pyramid Lake museum	0.297	0.230	0.094	0.014	
Winnemucca Lake mus	0.383	0.322	0.179	0.097	0.175

#### 3.3.2. Truckee River basin: Pilot Peak, Independence Lake and museum samples

**Figure 6.**
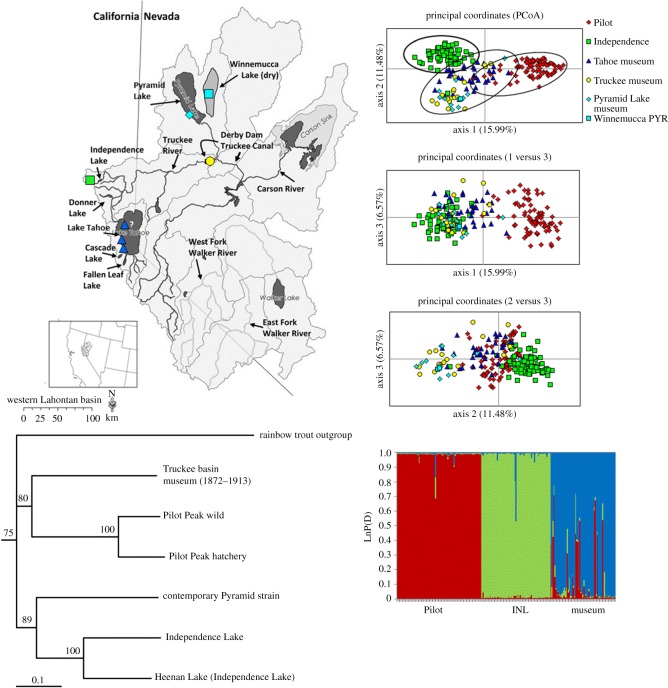
The results of Bayesian genotype clustering analysis, PCoA, and a Cavalli-Sforza genetic distance and neighbour-joining phylogenic analysis of the Truckee River historical museum samples, extant Pilot Peak and Independence Lake populations. Three genotype clusters are the best fit of the data, showing the Pilot and museum sharing membership in the red cluster and a little overlap with the Independence Lake cluster (green) (LnP(D) = −3513.3, *k *= 3, Δ*k*= 596.8). Sampling locations are colour coded in the map to correspond with PCoA and Bayesian plots.

Pairwise *F*_ST_ among Pilot, Independence and each of the museum sampling locations were also significant (*p* = 0.01, 99 permutations, table 4*b*). However, not surprisingly the *F*_ST_ estimates among the museum sites had the lowest values (table 4*b*). The Bayesian genotype clustering results reveal three genotype clusters (LnP(D) = −3513.3, Δ*k *= 596.8) with the museum samples being assigned primarily to two genotype clusters (blue and red) and all Pilot Peak strain individuals being assigned to the red genotype cluster (figure 6). The Independence Lake individuals assigned primarily to the green genotype cluster with one individual having some assignment to the blue museum cluster. Similarly, the PCoA results show overlap among these three groups (Museum, Independence and Pilot Peak), but more overlap between the museum samples and the Pilot Peak strain was observed compared with Independence Lake for axes 1 and 2, which account for more of the variance (figure 6). The topology of the Cavalli-Sforza neighbour-joining tree places the museum samples in a clade with the Pilot Peak strain (80% bootstrap value) separately from the Independence Lake trout, which form a clade with the contemporary Pyramid Lake strain (89% bootstrap value, figure 6).

#### 3.3.3. Truckee River basin museum samples

**Figure 7.**
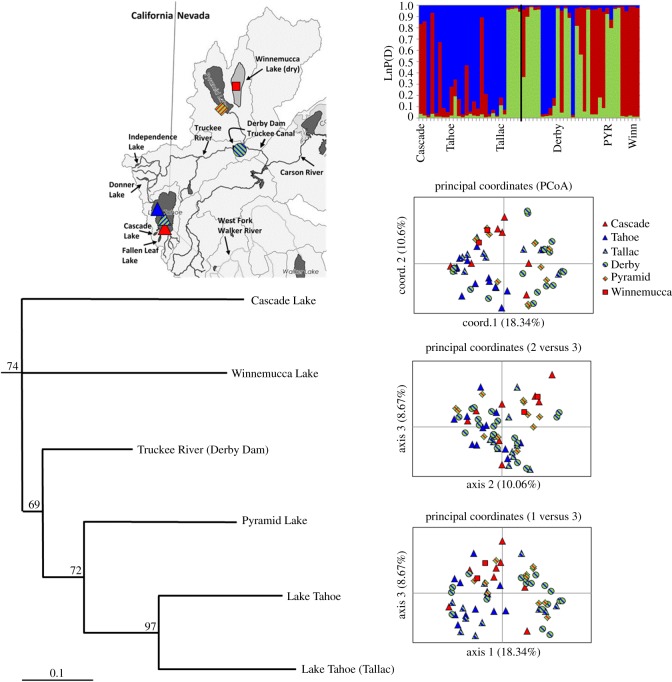
Results of Bayesian genotype clustering analysis (LnP(D) = –1059.7, Δ*k* = 104.8), PCoA and Cavalli-Sforza genetic distance and neighbour-joining phylogeny for historical museum samples only. Bayesian and PCoA results indicate some movement among sampling locations historically. The phylogenetic tree shows sites within Lake Tahoe more closely related to each other than to sites lower in the watershed. Interestingly, Cascade Lake appears to be more similar to individuals lower in the watershed than to closer sites in Lake Tahoe.

Genetic variance within the museum samples alone was partitioned as 69% within individuals, 26% among individuals, 4% among sampling locations and 1% among three regions (Lake Tahoe, Truckee River and Pyramid Lake). Nm estimates among the museum sites also suggest gene flow between the lower river and Pyramid Lake (Nm = 58) with additional limited gene flow among Tahoe, the lower river and Pyramid Lake (Nm = 4, 2.5, respectively). Among the museum sampling sites, three genotype clusters had the best statistical support, which roughly corresponds to the three sampling regions (LnP(D) = –1059.7, Δ*k* = 104.8; figure 7). However, both the Bayesian genotype clustering and PCoA suggest historical movement of individuals within the Truckee River basin such that membership in genotype clusters was not spatially organized according to sampling location (figure 7). The topology of the Cavalli-Sforza neighbour-joining tree also reveals differentiation within the basin. The Lake Tahoe museum collection sites, Tahoe and Tallac, are more closely related to each other than to other sampling locations, with Pyramid Lake being the next most closely related and then Truckee River site, Derby Dam. However, the Cascade Lake samples, which are in the Lake Tahoe basin, were more similar to Winnemucca Lake than they are to other Lake Tahoe samples, but this could be due to the limited sampling (*N* = 2) from Winnemucca Lake.

## 4. Discussion

The lacustrine lineage of Lahontan cutthroat trout historically found in Lake Tahoe and Pyramid Lake was the largest of the inland cutthroat trout subspecies and after the 1940s was believed to be extinct. The genetic results presented here together with morphological, meristic, growth and behavioural data suggest this is not the case. Genetic analyses support the hypothesis that Lahontan cutthroat trout found in an out-of-basin stream in the Pilot Peak Mountains of Utah are the original lacustrine strain native to the Truckee River basin in the western Lahontan hydrograph basin. However, we also acknowledge the uncertainty inherent in microsatellite data and the difficulty of working with preserved museum samples. That said, this population is the most genetically similar to the Lake Tahoe and Pyramid Lake museum specimens collected in the late ninenteenth and early twentieth centuries prior to the extirpation of these large lake populations, when compared with the majority of extant LCT populations, including Independence Lake, the only other extant population in the Truckee River basin.

Furthermore, genetic analyses indicate population genetic structure as well as movement within the basin historically, with Independence Lake supporting a largely distinct subpopulation. Although results from the Independence Lake samples, collected approximately 100 years later than the museum samples, may reflect the effects of isolation and drift in the contemporary Independence Lake population, we did observe comparable levels of allelic richness and heterozygosity per locus between the historic museum samples and contemporary Independence Lake samples. Although bottlenecks have certainly been an issue for Lahontan cutthroat trout populations in the twentieth century due to human perturbations, the museum samples were collected when this area was still sparsely populated and we found no evidence for a genetic bottleneck in the contemporary Independence Lake population. These data suggest the Independence Lake population has maintained genetic variation despite its current isolation and that the genetic differentiation observed probably reflects historical patterns. The Truckee River basin appears to have once supported multiple subpopulations of Lahontan cutthroat trout. Support for this interpretation is based on the observed patterns of population genetic structure and gene flow among extant subpopulations of this subspecies found in the few remaining large, multiple-order interconnected streams systems in the Humboldt River basin [36,38,50].

These results further support anecdotal information of anthropogenic movement of trout from Lake Tahoe and Pyramid Lake into waters in the eastern Lahontan basin and other out-of-basin waters such as fishless Morrison Creek in the Pilot Peak Mountains in Utah in the early part of the twentieth century. Given the genetic data together with the morphological and meristic characters and evidence of piscivory, which support a lacustrine life history as well as the record growth rates and large sizes being achieved in Pyramid Lake today, the Pilot Peak strain represents an important part of the lacustrine legacy of this subspecies of cutthroat trout.

Under an ESU framework, native fishes should be used to restore populations in historical habitat. Implicit in this aim is that locally adapted fishes have a greater chance of re-establishing populations [19,20]. Owing to morphological and meristic differences between trout native to the lacustrine habitats of western and northwestern Lahontan basins and the Humboldt River system (e.g. Humboldt fish have fewer gill rakers, fewer pyloric caeca and tend to have fewer scales in the lateral series and above the lateral line; [25]), Behnke [16] and Trotter and Behnke [51] have proposed that the Lahontan subspecies be split into separate Lahontan and Humboldt (*O. clarkii* subsp.) subspecies, which would better reflect the lacustrine and fluvial life histories of these fish and be consistent with the observed morphological differences.

The use of the Pilot Peak strain of Lahontan cutthroat trout in recovery activities in the large lacustrine ecosystems of the western Lahontan basin ESU offers a rare opportunity to not only restore a native lineage to its historical waters, but in doing so also recover the breadth of life-history strategies and the suite of adaptations these fish may have had to these large lacustrine habitats.

## 5. Conclusion

In the ninenteenth and early twentieth centuries, museums worldwide created natural history collections for a diverse array of taxa in order to catalogue and voucher species as they were discovered. For over 500 years, such collections have been used to examine local and regional patterns of morphological variation. In just the past 20 years, however, improved DNA extraction methodologies and new analytical techniques have been applied to these specimens, allowing researchers to place patterns of genetic variation in a historical context [52]. As techniques continue to improve, data recovery is allowing researchers to compare temporal trends in genetic variation and genetic structure of extant organisms in the context of anthropogenic perturbation [53–59]. Most recently, genetic analyses have been used to characterize genetic variation in long extinct species [41,60–63]. However, such studies are only possible where collections are available.

In North American collections, specimens from both aquatic and terrestrial vertebrates, although relatively abundant, are at continued risk due to budgetary and storage constraints [64]. This study once again highlights the value of these collections to empirically assess baseline patterns of genetic connectivity and to measure the distribution of unique genetic variants in vanishing species. The genetic analysis of archival museum specimens offers an opportunity to examine how organisms assorted themselves on the landscape historically, which may give insight into past population dynamics and inform restoration strategies that capture natural dynamics and increase the probability of successful recovery of threatened and endangered species [58].

## Data accessibility

We have deposited the genetic data (microsatellite genotypes) for this paper in Dryad: (http://dx.doi.org/10.5061/dryad.24cc7) [65].
